# TIPE3 differentially modulates proliferation and migration of human non-small-cell lung cancer cells via distinct subcellular location

**DOI:** 10.1186/s12885-018-4177-0

**Published:** 2018-03-06

**Authors:** Guannan Wang, Chun Guo, Hui Zhao, Zhenzhen Pan, Faliang Zhu, Lining Zhang, Qun Wang

**Affiliations:** Department of Immunology and Key Laboratory of Infection and Immunity of Shandong Province, School of Basic Medical Sciences, Shandong University, Jinan, Shandong 250012 China

**Keywords:** TIPE3, Non-small-cell lung cancer, Proliferation, Migration

## Abstract

**Background:**

TIPE3 (TNFAIP8L3), a transfer protein for lipid second messengers, is upregulated in human lung cancer tissues. The most popular lung cancer is non-small cell lung cancer (NSCLC) with high incidences and low survival rates, while the roles of TIPE3 in NSCLC remain largely unknown.

**Methods:**

TIPE3 expression was examined in tissue chips from patients with NSCLC using immunohistochemistry; the correlation of plasma membrane expression of TIPE3 with T stage of NSCLC was analyzed. After endogenous TIPE3 was silenced via siRNA, or TIPE3 with N or C-terminal flag was overexpressed via transient or stable transfection, human NSCLC cells were assayed for the proliferation and migration, respectively. NSCLC cells, in which TIPE3 with C-terminal flag was stably transfected, were inoculated into mice to establish xenograft tumors, the tumor growth and the expression of TIPE3 in tumor tissues were examined.

**Results:**

TIPE3 was broadly expressed in lung tissues of patients with NSCLC. The plasma membrane expression of TIPE3 was positively correlated with the T stage of NSCLC. Knockdown of endogenous TIPE3, which was predominantly expressed in the plasma membrane, inhibited the proliferation and migration of NSCLC cells. While transient overexpression of TIPE3 with N-terminal flag, which was mostly trapped in the cytoplasm, inhibited the growth and migration of NSCLC cells accompanied by inactivation of AKT and ERK. In contrast, stable overexpression of TIPE3 with C-terminal flag, which could be localized in the plasma membrane, markedly promoted the growth and migration of NSCLC cells through activation of AKT and ERK. Notably, in xenograft tumor models established with NSCLC cells, stable overexpression of TIPE3 with C-terminal flag in NSCLC cells significantly promoted the tumor growth and enhanced the expression and plasma membrane localization of TIPE3 in tumor tissues.

**Conclusion:**

This study demonstrates that human TIPE3 promotes the proliferation and migration of NSCLC cells depending on its localization on plasma membrane, whereas cytoplasmic TIPE3 may exert a negative function. Thus, manipulating the subcellular location of TIPE3 can be a promising strategy for NSCLC therapy.

**Electronic supplementary material:**

The online version of this article (10.1186/s12885-018-4177-0) contains supplementary material, which is available to authorized users.

## Background

Lung cancer is the leading cause of cancer death in males, and is the second cause of cancer death in females worldwide [1]. The incidence and mortality of lung cancer are increasing rapidly in recent years, which are mainly caused by environmental pollution and smoking. Non-small cell lung cancer (NSCLC) accounts for about 85% of all lung cancers, and can be any type of epithelial lung cancer other than small cell lung cancer. The most common types of NSCLC include squamous cell carcinoma, large cell carcinoma, and adenocarcinoma. Although there are great developments in NSCLC treatment, patients with advanced NSCLC still have a very low five-year survival rates [2–5]. Therefore, it is of great importance to identify the potential molecular targets for NSCLC therapy.

TIPE3 is the newest member of TIPE (tumor necrosis factor-α-induced protein 8, TNFAIP8) family, which consists of a group of proteins including TIPE, TIPE1 and TIPE2 that regulate tumorigenesis and immunity [6–11]. TIPE3 gene locates human chromosome 15 or murine chromosome 9 [12–14]. TIPE3 protein is extensively expressed in murine organs including uterus, lung, brain, bladder, intestine and colon with various expression levels. Notably, marked increase in TIPE3 expression was detected in human cancer tissues including cervical, colon, lung and esophageal. Murine TIPE3 has been demonstrated to serve as a phosphoinositide carrier to activate PI3K-AKT and MEK-ERK signaling pathways, thus promoting the growth and tumorigenesis of NIH3T3-HRasV12 cells [6, 8]. Similar to other family members, TIPE3 has a highly conserved TIPE2 homology (TH) domain. The crystal structure showed that the α1-α6 helixes of human TIPE3 TH-domain formed a large hydrophobic cavity, which likely accommodates lipid secondary messengers PIP2 and PIP3 for phosphoinositide signaling [8]. TIPE3 expression was found in normal human lung tissues, and markedly increased in human lung cancer tissues [8, 15]. It remains unclear whether and how TIPE3 is involved in human NSCLC. In the present study, we revealed that plasma membrane-localizing TIPE3 was positively correlated with the malignance of NSCLC. Using two different NSCLC cell lines and xenograft tumor models, we demonstrated that endogenous human TIPE3 promoted cell proliferation and migration in NSCLC; while exogenous human TIPE3 produced differential roles in the growth and migration of NSCLC cells based on its different subcellular location.

## Methods

### Patients

Lung tissue arrays from 48 cases of human primary NSCLC (OUTDO BIOTECH, Shanghai, China) were used to detect the expression of TIPE3. The correlation between TIPE3 expression and clinical features, as well as the correlation of plasma membrane expression of TIPE3 with T stage of NSCLC were analyzed.

### Cell culture

The H1975 cell line (human lung adenocarcinoma) (TCHu193) was purchased from Shanghai Cell Bank of Chinese Academy of Sciences (Shanghai, China), and cultured in RPMI 1640 medium (Gibco, CA, USA) containing 10% fetal bovine serum (FBS) (Gibco). The A549 cell line (human lung adenocarcinoma) (GDC063) was purchased from China Center for Type Culture Collection (Wuhan, China), and cultured in F12 K medium (Macgene, Beijing, China) supplemented with 10% FBS.

### Plasmids, siRNA and transfection

Plasmid carrying N-terminal flag-tagged human TIPE3 genes was constructed by Sangon Biotech (Shanghai, China). Specific siRNA for human TIPE3 (5’CGCAGCAUGGAUUCGGAUUdTdT3’, 3’dTdTGCGUCGUACCUAAGCCUAA5’; 5’GGAACGUGCUCUCCAAUCUdTdT3’, 3’dTdTCCUUGCACGAGAGGUUAGA5’) was designed and synthesized by RIBOBIO (Guangzhou, China). Tumor cells were transfected with plasmid or siRNA using Lipofectamine 2000 (Invitrogen, Carlsbad, CA) according to the manufacturer’s protocols. Recombinant lentiviral vector carrying C-terminal flag-tagged human TIPE3 gene was constructed by Genechem (Shanghai, China). Lentiviral transfection was performed in tumor cells as per the instruction.

### Reverse transcriptional PCR

Total RNA was extracted from cells using Trizol Reagent (Invitrogen). PCR was performed using 2 × Taq PCR MasterMix (TIANGEN, Beijing, China). The primers were as follows: human TIPE3, 5′-GAGGAGCTGGTTATTGTGGAGAA-3′ and 5′-ATCGGCAAAGTGGTTAAAGACG-3′; human GAPDH, 5′-AACGGATTTGGTCGTATTGGG-3′ and 5′-CCTGGAAGATGGTGATGGGAT-3′.

### Western-blot

Equal amount of protein was separated by SDS-PAGE and transferred onto PVDF membranes (Millipore, Billerica, MA). Membranes were probed overnight at 4 °C with primary antibodies against human TIPE3 (1:300; BOSTER, Wuhan, China), p-AKT, p-ERK, AKT, ERK (1:1000, Cell Signaling Technology, Beverly, MA), and flag (1:10000, MBL, Nagano, Japan), GAPDH or β-actin (1:1000; ZSGB-Bio, Beijing, China), followed by secondary antibodies (1:2000; ZSGB-Bio) conjugated with peroxidase for 1 h at room temperature. Signals were detected by SuperSignal West Pico Chemiluminescent Substrate (Pierce Biotechnology, Rockford, IL).

### Immunohistochemistry

The paraffin slides were stained with rabbit antibody against human TIPE3 (1:200) at 4 °C overnight, followed by HRP-conjugated anti-rabbit IgG using MaxVsion Kit. The 3, 5-diaminobenzidine peroxidase Substrate Kit (Maixin, Fuzhou, China) was used for color detection. The sections were counterstained with hematoxylin. The results were independently assessed by two experienced pathologists. The staining intensity was scored from 0 to 3 (0, no staining; 1, weak; 2, moderate; 3, strong). The staining extent was scored from 0 to 3 based on the percentage of positive cells (0, < 1%; 1, 1%–33%; 2, 34%–66%; 3, 67%–100%). The two scores for each slide were then combined to produce a final grade of TIPE3 expression: 0–2, low; 3–8, high. The average score was used if there were discrepancies in the assessment.

### Immunofluorescence

The cells on cover slips were fixed, permeabilized and then were probed with rabbit antibody against human TIPE3 (1:100) and mouse antibody against α1 **Na**^**+**^**/K**^**+**^**-**ATPase (1:200; abcam, Cambridge, UK) overnight at 4 °C, followed by Alexa Fluor 594-conjugated goat anti-rabbit IgG (1:300, Proteintech Group, Rocky Hill, NJ) and FITC-conjugated goat anti-mouse IgG (1:300, CWBIO, Beijing, China). Nuclei were stained by 4′, 6-diamidino-2-phenylin-dole (DAPI) (Beyotime, Shanghai, China) for 5 min. Results were analyzed on confocal laser microscopy (Carl Zeiss, LSM780, Oberkochen, Germany).

### Cell viability assay

Cells were seeded in 96-well plates at 3000 cells per well and cultured for indicated time periods. Cell viability was evaluated using CCK8 (DOJINDO LABORATORISE, Japan) according to the manufacturer’s instructions. The absorbance was determined at 450 nm.

### Cell migration assay

Tumor cell migration was analyzed in 24 well Boyden chambers with 8-μm pore size polycarbonate membranes (Costar, Acton). Cells (4 × 10^4^) were suspended in 200 μl serum-free medium and placed in the upper chamber. The lower compartments were filled with 600 μl medium with 10% FBS. After 10 h of incubation, the cells remaining on the upper surface of the membrane were removed. The cells on the lower surface of the membrane were fixed and stained with crystal violet, and then were counted under light microscope at × 200 magnification.

### Establishment of xenograft tumors in nude mice

Male BALB/c nu/nu mice (4–6 week old) were purchased from Chinese Academy of Sciences (Shanghai, China) and maintained in laminar-flow cabinets under specific pathogen-free conditions. A549 cells transfected with mock or recombinant lentivirus were subcutaneously injected into flanks of nude mice. The tumor growth was monitored by calculating the tumor volume (length×width×width). The mice were sacrificed under anesthesia 30 days after inoculation. The tumors were resected and processed for standard histopathological study. All procedures were approved by Ethical Review Board of Shandong University, and carried out in accordance with the Animal [Scientific Procedures] Act 1986 and the institutional guidelines for animal care and utilization.

### Statistical analysis

Statistical analysis was performed with GraphPad Prism 5. Chi-square or Fisher’s exact test was used to evaluate correlation; Student’s *t* test, One-way or Two-way ANOVA were used to evaluate differences. *P* value < 0.05 was considered statistically significant.

## Results

### TIPE3 localized in plasma membrane positively correlates with T stage in patients with NSCLC

**Fig. 1:**
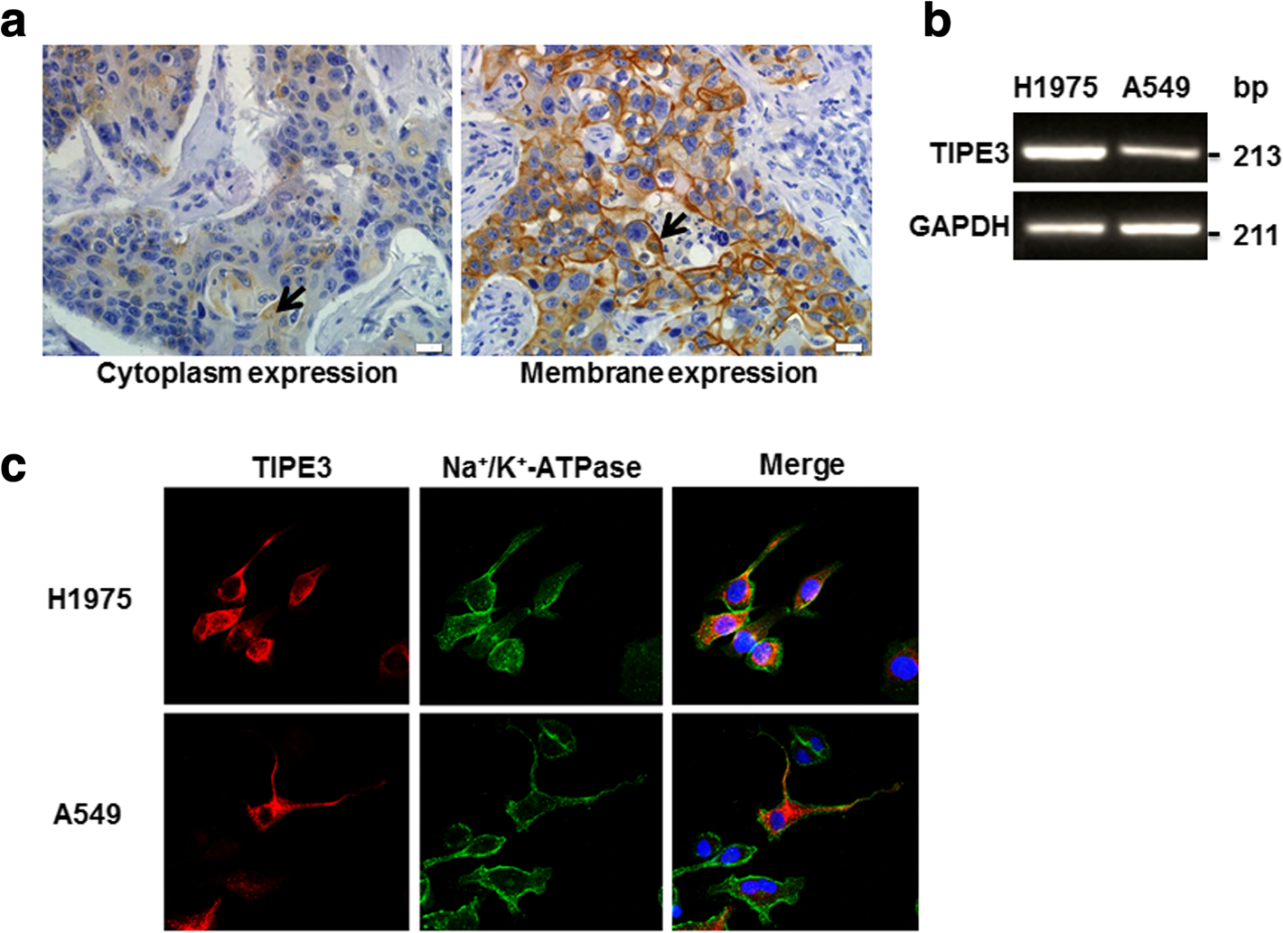
Expression of TIPE3 on tissues and cell lines from NSCLC. **a** TIPE3 expression was detected on lung tissue chips from patients with primary NSCLC by immunohistochemistry. **b** The mRNA of TIPE3 in NSCLC cell lines H1975 and A549 were detected by PCR. **c** The expression of endogenous TIPE3 and **Na**^**+**^**/K**^**+**^**-**ATPase was detected using confocal laser microscopy

It has been recognized that mouse TIPE3 serves as a transfer protein for lipid second messengers to promote cancers [8], whereas the potential roles of human TIPE3 in NSCLC remain to be clarified. We showed that TIPE3 was broadly expressed in cancer tissues of patients with NSCLC. There was no correlation between TIPE3 expression and clinical features including age, gender, T stage and pathological grade (*p* > 0.05) (Table 1). However, in some samples TIPE3 was pervasively expressed in the cytoplasm of cancer cells, while in others TIPE3 was mainly expressed in the plasma membrane of cancer cells (Fig. 1a). We further analyzed the correlation between subcellular localization of TIPE3 and T stage. Interestingly, the expression of TIPE3 on plasma membrane was positively correlated with the T stage of NSCLC (*p* < 0.05) (Table 2), indicating that TIPE3 located in plasma membrane may exert pro-tumorigenic activity.

**Table 1:** Relation of TIPE3 expression and clinical parameters in patients with NSCLC

Characteristic	TIPE3		*P* value
	Low(0~ 2)	High(3~ 8)	
Gender			
Male	5	24	0.096
Female	8	11	
Age(years)			
< 60	7	16	0.749
≥ 60	6	19	
T Stage			
T1	3	7	0.999
T2-T3	10	28	
Pathological grade			
I-II	1	7	0.418
II-III	12	28	

(I-II: I and I-II; II-III: II, II-III and III)

**Table 2:** Association of subcellular location of TIPE3 with T stage in patients with NSCLC

Characteristic	TIPE3		*P* value
	Plasma membrane	Cytoplasm	
T stage			
T1	3	4	0.049
T2	17	3	

### Endogenous TIPE3 gathers in the plasma membrane of lung cancer cells with high viability

To clarify the function of TIPE3 in NSCLC, H1975 and A549 cells (NSCLC cell lines) were used to detect the expression and subcellular location of endogenous TIPE3. Higher levels of TIPE3 mRNA were detected in H1975 cells compared with A549 cells (Fig. 1b). Similar to lung cancer tissues, TIPE3 expression was observed in cytoplasm as well as the inner side of plasma membrane in both H1975 and A549 cells. Almost all of H1975 cells expressed TIPE3, which  was  mainly localized in plasma membrane. Differently, only part of A549 cells expressed TIPE3, in which plasma membrane-localizing TIPE3 was mainly expressed in cells with long and multiple pseudopodia, whereas cytoplasm-localizing TIPE3 was mostly expressed in cells with less pseudopodium. In particular, strong expression of TIPE3 was detected on protrusion of both H1975 and A549 cells (Fig. 1c), suggesting the potential link between the plasma membrane expression of TIPE3 and the viability of cancer cells.

### Silence of endogenous TIPE3 attenuates the proliferation and migration of lung cancer cells

**Fig. 2:**
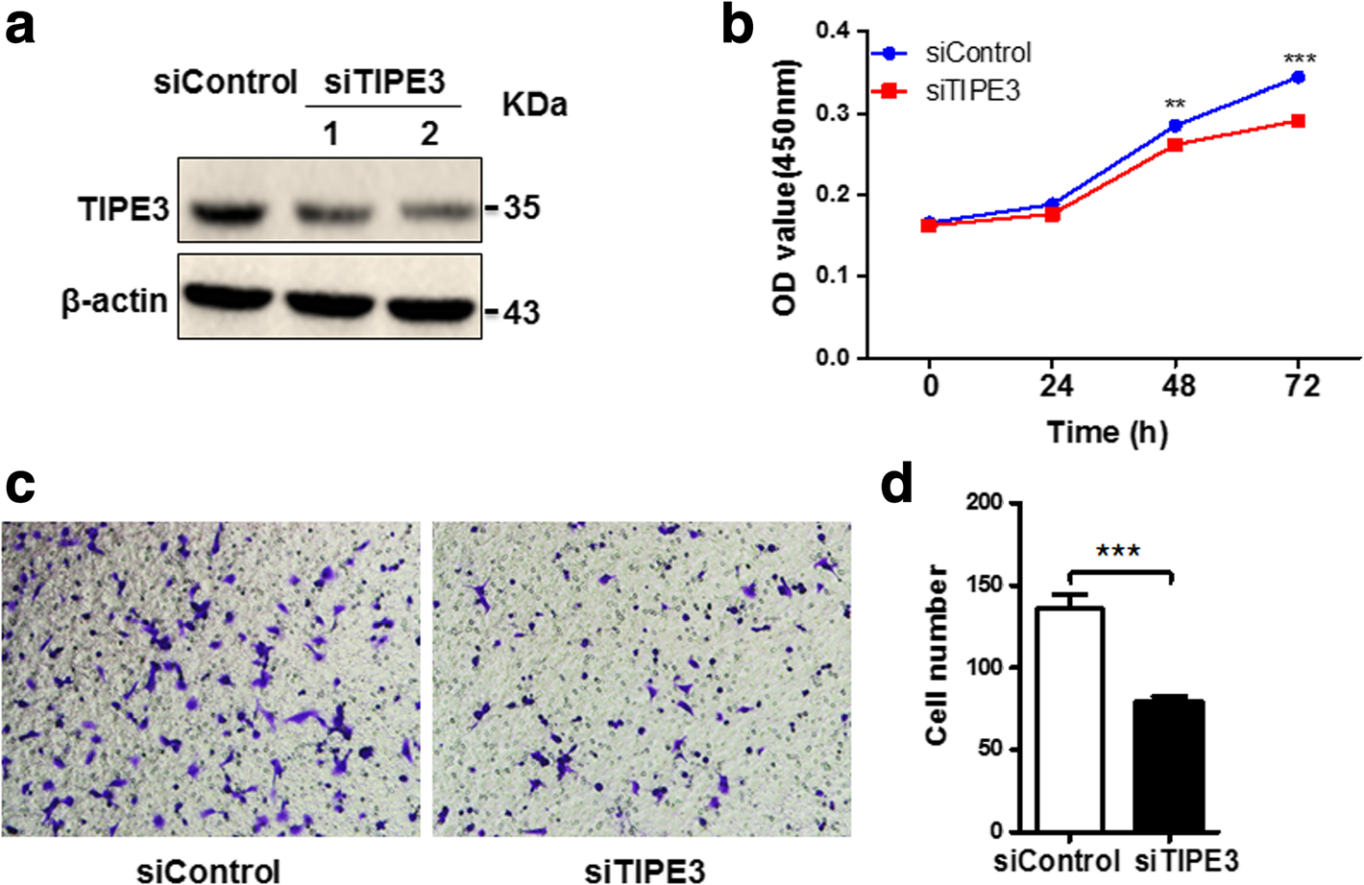
Silence of endogenous TIPE3 attenuates the proliferation and migration of lung cancer cells. **a** Endogenous TIPE3 was knocked down in H1975 cells using siTIPE3. **b** The growth curve of H1975 cells after silence of TIPE3 was determined by CCK8 assay (*n* = 4 per timepoint). **c, d** The migration of H1975 cells after silence of TIPE3 was determined. Representative (**c**) and statistic (**d**) data are shown (*n* = 5 per group). Bars represent mean ± s.e.m. **P < 0.01, ***P < 0.001

To clarify the effects of TIPE3 on the proliferation and migration of lung cancer cells, we used siTIPE3 to knock down the expression of endogenous TIPE3 in H1975 cells, which expressed higher level of TIPE3 than A549 cells (Fig. 2a). After transfection with siTIPE3, H1975 cells showed a marked growth inhibition at 48 h or 72 h (Fig. 2b). Accordingly, the migration of H1975 cells was also inhibited by silencing endogenous TIPE3 (Fig. 2c and d). These data demonstrate that endogenous TIPE3 plays promotive effects on the proliferation and migration of lung cancer cells.

### Exogenous TIPE3 located in cytoplasm inhibits the proliferation and migration of lung cancer cells

**Fig. 3:**
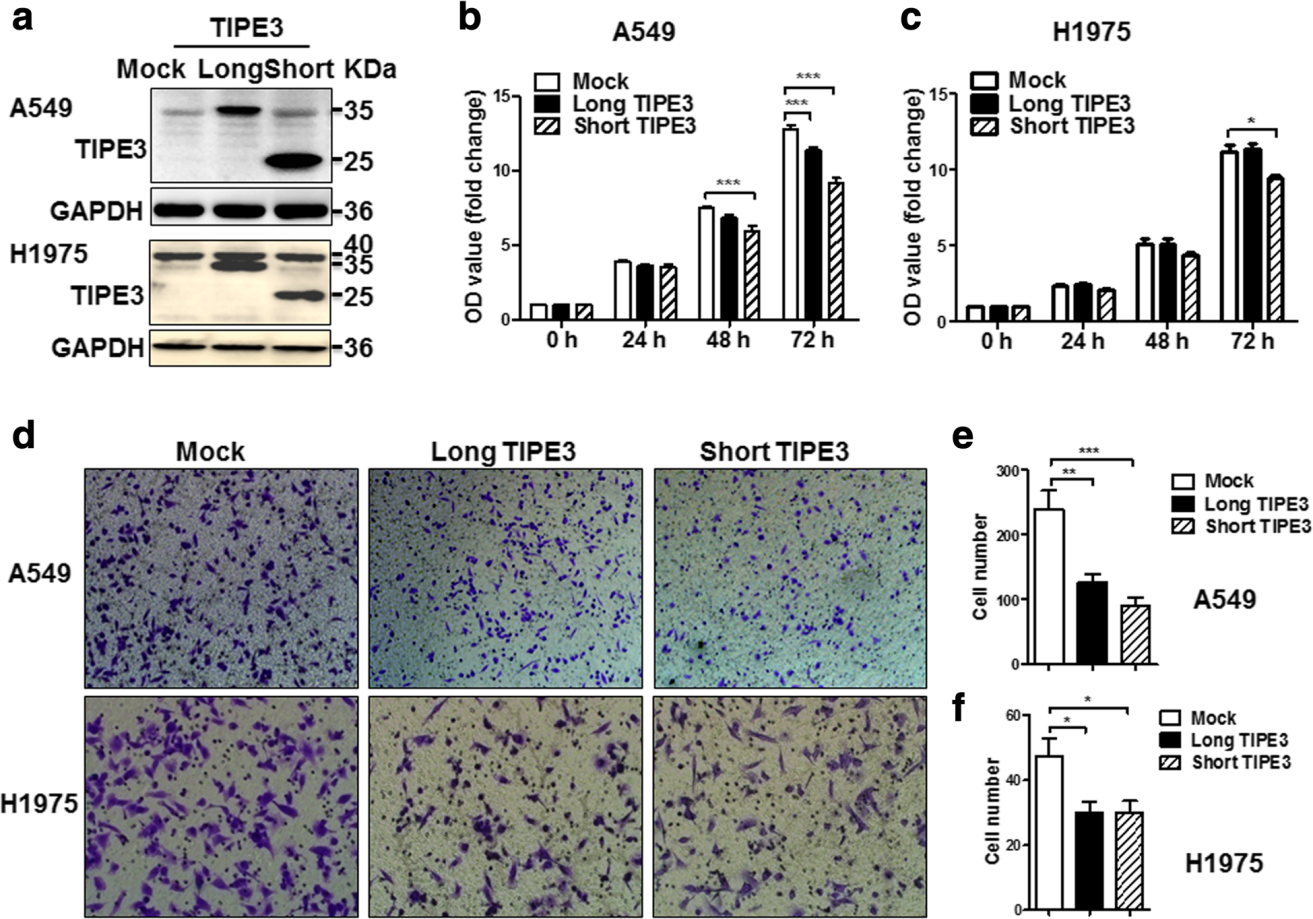
Overexpression of TIPE3 with N-terminal flag inhibits the proliferation and migration of lung cancer cells. **a** Long or short TIPE3 with N-terminal flag was transiently overexpressed in A549 and H1975 cells by plasmid transfection, and detected by western-blot. **b, c** The growth curves of A549 (**b**) and H1975 (**c**) cells overexpressing TIPE3 were determined by CCK8 assay (*n* = 4 per timepoint). **d-f** The migration of A549 and H1975 cells overexpressing TIPE3 was determined. Representative (**d**) and statistic (**e, f**) data are shown (*n* = 5 per group). Bars represent mean ± s.e.m. *P < 0.05, **P < 0.01, ***P < 0.001

Since there are two transcript variants of human TIPE3, we constructed two plasmids overexpressing human TIPE3 with N-terminal flag, one full-length human TIPE3 (long TIPE3) and another short human TIPE3 (short TIPE3) highly similar to mice TIPE3 (Supplementary Material, Additional file 1). After transfection with these two plasmids respectively, both A549 and H1975 cells successfully expressed long or short TIPE3 (Fig. 3a). To our surprise, the growth and proliferation of A549 cells were significantly inhibited by overexpressing either long or short TIPE3 (Fig. 3b); the growth of H1975 cells was inhibited by overexpressing short TIPE3 but not long TIPE3 (Fig. 3c). In cell migration assay, overexpression of either long or short TIPE3 led to an inhibition in the migration of both A549 and H1975 cells (Fig. 3d-f). These findings indicate that transient expression of exogenous TIPE3 via plasmid transfection inhibits the growth and migration of lung cancer cells.

**Fig. 4:**
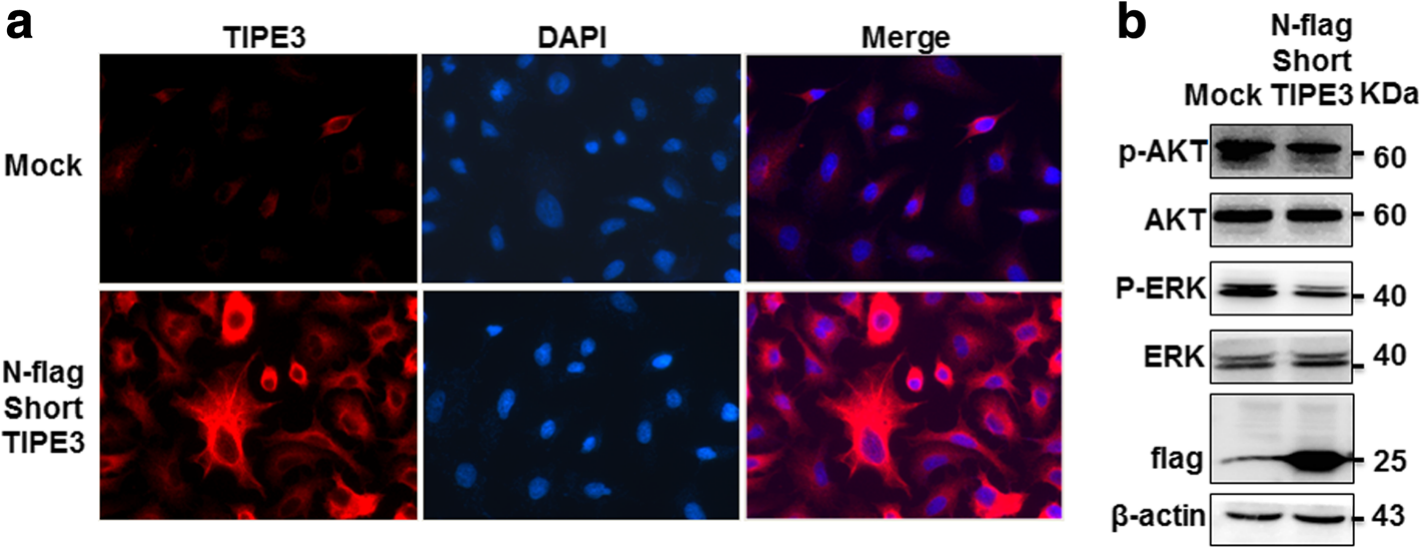
Exogenous TIPE3 with N-terminal flag is mainly located in the cytoplasm of lung cancer cells. Exogenous short TIPE3 with N-terminal flag was transiently overexpressed in A549 cells by plasmid transfection. **a** The subcellular location of TIPE3 was detected using confocal laser microscopy. **b** The levels of p-AKT and p-MEK were determined by western-blot

To elucidate the possible reasons for the inhibitory effects of exogenous TIPE3 on cell proliferation and migration, we performed immunofluorescence to detect the subcellular localization of exogenous short TIPE3. We found that A549 cells transfected with TIPE3-expresing plasmid showed strong expression of TIPE3 with predominant location in the cytoplasm, even in vigorous cells with many pseudopodia. In contrast, plasma membrane localization of TIPE3 was rarely found (Fig. 4a). Since the activation of AKT and ERK contributes to cell survival and growth, we further assayed and verified marked inhibitions in the levels of both p-AKT and p-ERK (Fig. 4b), indicating that transient overexpression of TIPE3 with N-terminal flag by plasmid transfection led to the cytoplasmic localization of exogenous TIPE3, which inhibited the proliferation and migration of lung cancer cells by inactivating AKT and ERK.

### Exogenous TIPE3 located in plasma membrane promotes the proliferation and migration of lung cancer cells

**Fig. 5:**
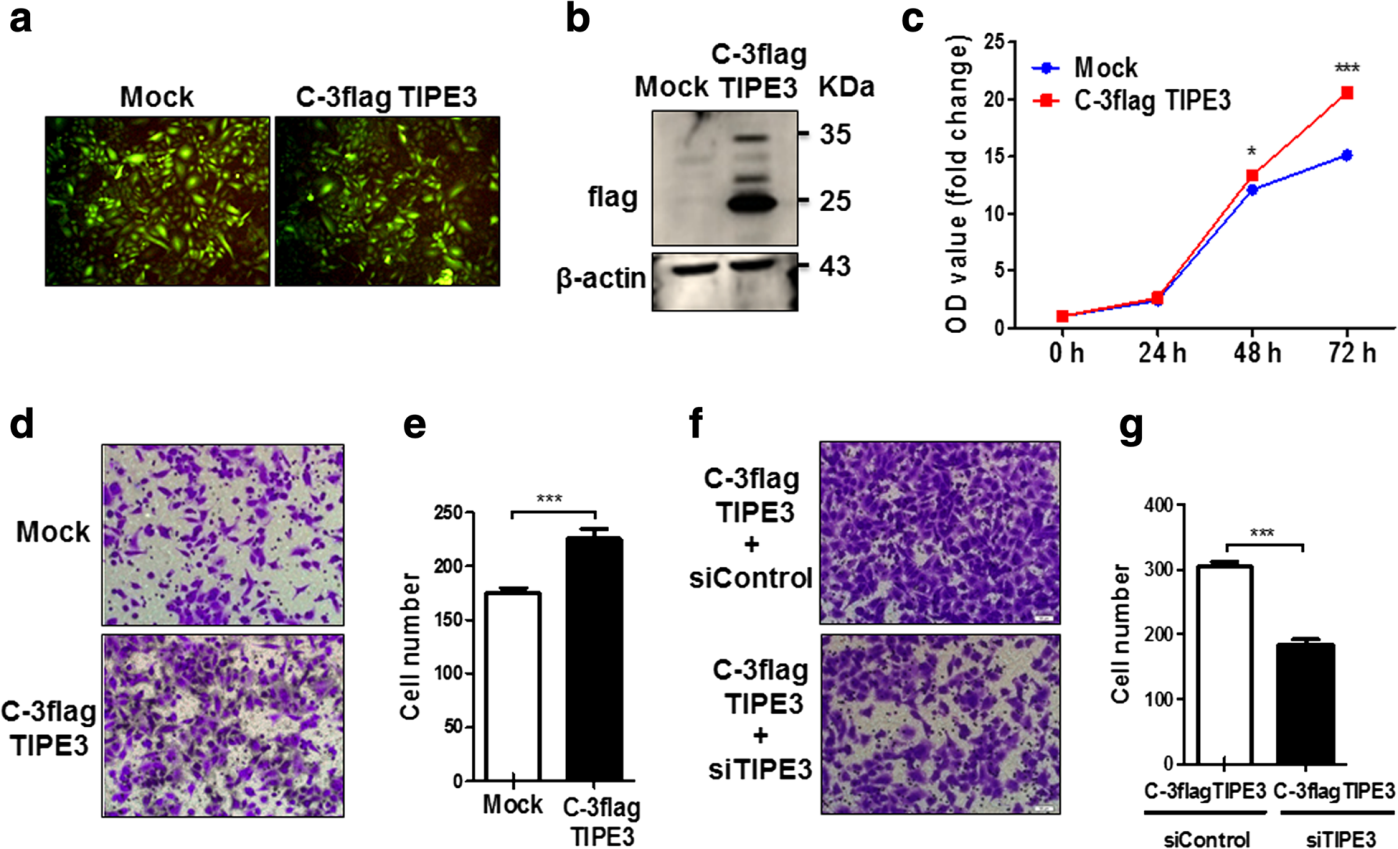
Overexpression of exogenous TIPE3 promotes the proliferation and migration of lung cancer cells. **a, b** TIPE3 with C-terminal flag was stably overexpressed in A549 cells by lentivirus transfection. GFP was detected by immunofluorescence (**a**), and flag was detected by western-blot (**b**). **c** The growth curves of A549 cells overexpressing TIPE3 were determined by CCK8 assay (*n* = 6 per timepoint). **d, e** The migration of A549 cells overexpressing TIPE3 was determined. Representative (**d**) and statistic (**e**) data (*n* = 5 per group) are shown. **f, g** The migration of A549 cells overexpressing TIPE3 was determined in the absence or presence of siTIPE3. Representative (**f**) and statistic (**g**) data are shown (*n* = 5 per group). Bars represent mean ± s.e.m. *P < 0.05, ***P < 0.001

**Fig. 6:**
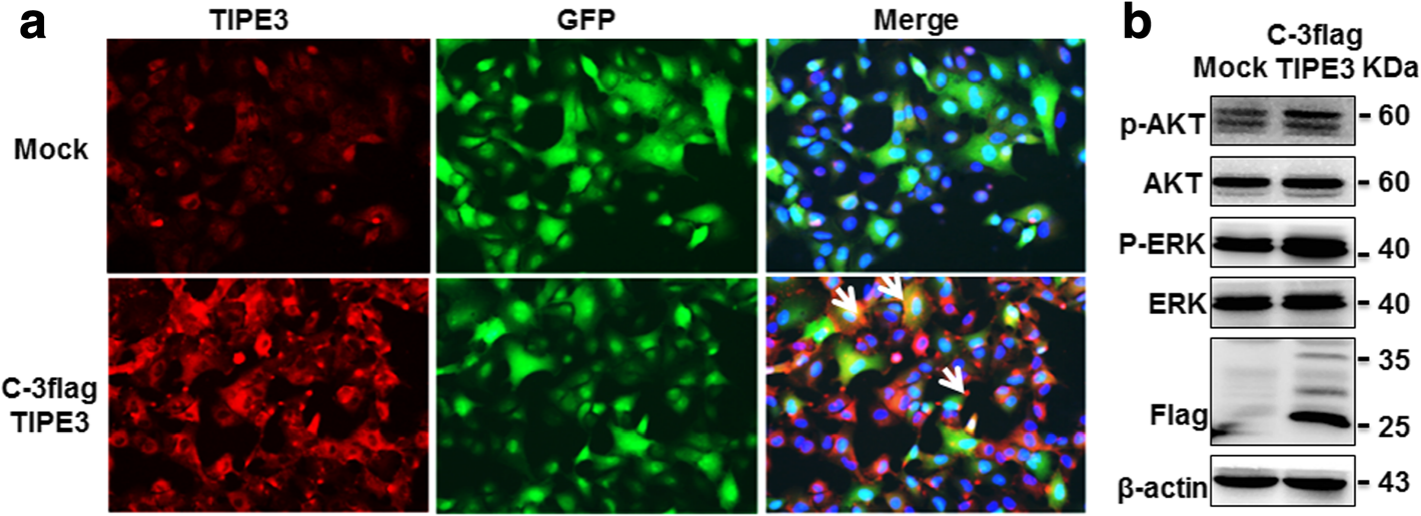
Exogenous TIPE3 with C-terminal flag is mainly located in the plasma membrane of lung cancer cells. Exogenous short TIPE3 with C-terminal flag was stably overexpressed in A549 cells by lentivirus transfection. **a** The subcellular location of TIPE3 was detected using confocal laser microscopy. **b** The levels of p-AKT and p-MEK were determined by western-blot

Considering the possible influence of N-terminal flag on the subcellular location and function of TIPE3, we further constructed recombinant lentivirus expressing human TIPE3 with C-terminal flag. Since both control and recombinant lentiviral vectors carried GFP genes, the presence of fluorescence signals in A549 cells confirmed the successful transfection by lentivirus (Fig. 5a). Flag protein was clearly detected in A549 cells transfected with recombinant lentivirus, indicating the stable expression of exogenous TIPE3 (Fig. 5b); accordingly, significant increases in growth and migration were detected in these cells (Fig. 5c-e). Next, knockdown of TIPE3 in A549 cells stably expressing exogenous TIPE3 caused a decrease in the ability of cell migration (Fig. 5f and g). These findings demonstrated that stable expression of exogenous TIPE3 through lentivirus transfection promoted the growth and migration of lung cancer cells. Further immunofluorescence confirmed that TIPE3 displaying dot-like fluorescence signals mainly gathered in the plasma membrane (Fig. 6a), indicating that exogenous TIPE3 located in plasma membrane is crucial for the proliferation and migration of lung cancer cells. It has been recognized that murine TIPE3 is involved in the activation of AKT and ERK pathway [8]. We further explored the levels of p-AKT and p-ERK, and verified their upregulation when human TIPE3 was located in the plasma membrane (Fig. 6b), indicating that human TIPE3 located in plasma membrane could function through AKT and ERK activation.

### Exogenous TIPE3 located in plasma membrane promotes the proliferation of lung cancer cells in vivo

**Fig. 7:**
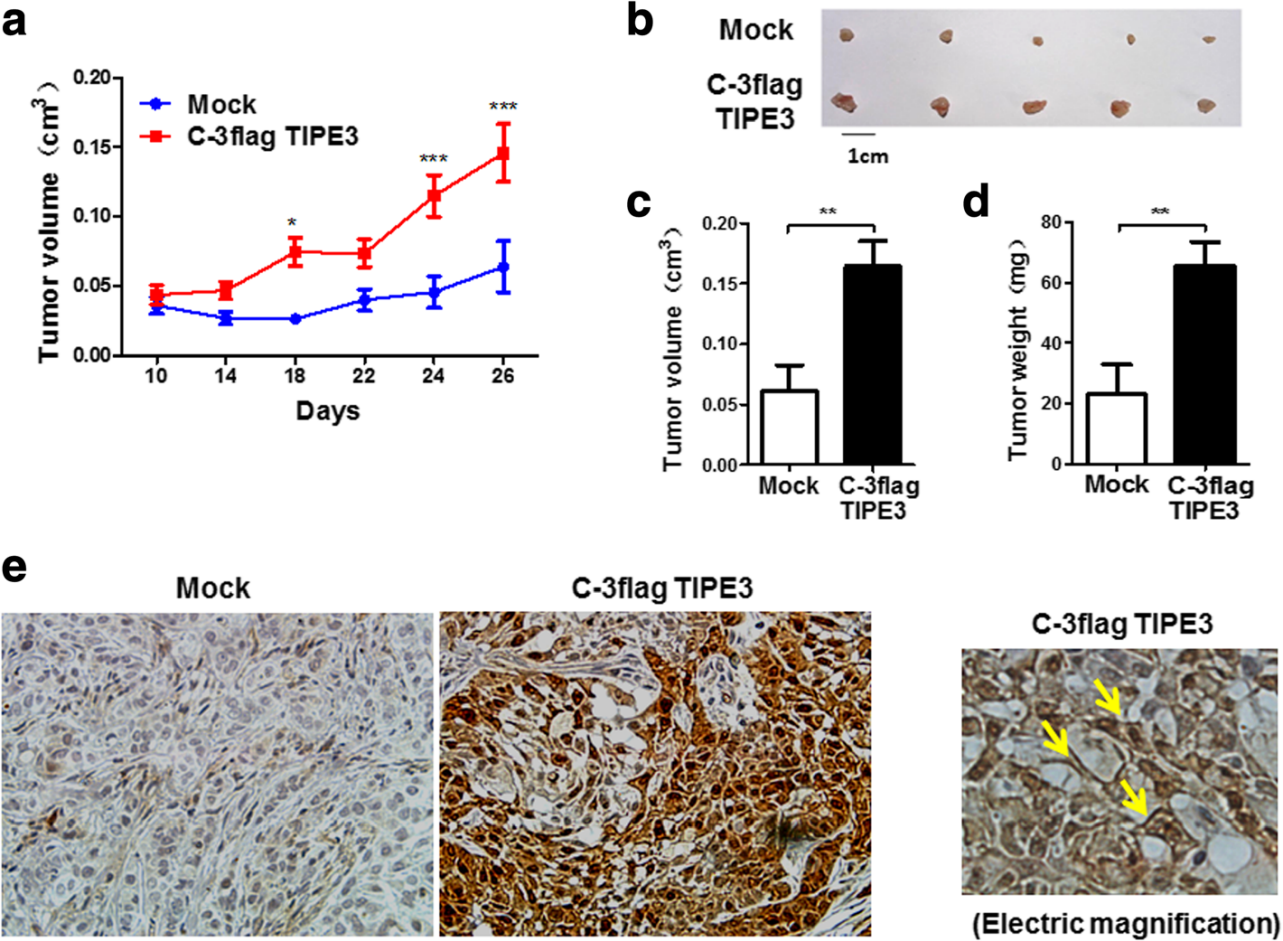
Exogenous TIPE3 with C-terminal flag promotes tumor growth in vivo. A549 cells (3 × 10^6^ cells in 100 μl PBS per mice) transfected with mock or recombinant (carrying C-terminal flag-tagged human TIPE3) lentivirus were subcutaneously injected into nu/nu mice (*n* = 5 per group) to establish xenograft tumors. **a-d** The growth curve of tumors (**a**), tumor size (**b, c**) and weight (**d**) were determined. **e** TIPE3 expression was evaluated in sections of tumor tissues by immunohistochemistry. *P < 0.05, ***P < 0.001

Next, we established subcutaneous xenograft tumor models in nude mice using A549 cells transfected with recombinant lentivirus expressing C-terminal flag-tagged human TIPE3. Mice received A549 cells overexpressing TIPE3 showed a marked increase in tumor growth, as confirmed by increased tumor volume and weight (Fig. 7a-d). Tumor tissues from mice implanted with TIPE3-transfected A549 cells showed enhanced TIPE3 levels compared with those from mice implanted with mock-transfected A549 cells, and importantly, most of TIPE3 was localized in the plasma membrane of tumor cells (Fig. 7e), indicating TIPE3 located in plasma membrane could promote the proliferation of lung cancer cells in vivo.

## Discussion

Human TIPE3 is highly expressed in lung cancer tissues compared with adjacent non-cancer tissues, indicating the possible involvement of TIPE3 in lung tumorigenesis [8, 15]. Considering high incidences and low survival rates of NSCLC, we tried to reveal the connection between TIPE3 and NSCLC. Through correlation analysis, we found no correlation of TIPE3 expression with clinical features in patients with NSCLC, but positive correlation of plasma membrane-localizing TIPE3 with T stage of NSCLC. These findings raise the possibility that human TIPE3 affects the tumorigenesis of NSCLC depending on its subcellular location.

Using two NSCLC cell lines A549 and H1975, we showed that TIPE3 was highly expressed in plasma membrane of the lung cancer cells with long pseudopodia, particularly in the position of protrusion. It has been recognized that protrusion formation is essential for cell migration to favor caner dissemination [16–18]. So, in the present study, TIPE3 on plasma membrane may be involved in cell motility and contribute to the growth and migration of lung cancer cells. Next, we showed that silence of endogenous TIPE3 significantly inhibited the growth and migration of NSCLC cells. Consistently, stably overexpression of exogenous TIPE3 via recombinant lentivirus transfection significantly promoted the growth and migration of NSCLC cells. In this stable overexpression system, we found that much TIPE3 scattered in the plasma membrane, although some TIPE3 still existed in the cytoplasm; simultaneously, the expression levels of p-AKT and p-ERK that is critical for cell survival, growth and migration [19–23], were obviously upregulated. These observations support previous finding in NIH3T3 cells that TIPE3 serves as a lipid transfer to activate PI3K-AKT and MEK-ERK pathways [8], and further suggest that TIPE3 may promote the growth and migration of lung cancer cells through AKT and ERK activation. To confirm the role and subcellular location of TIPE3 in the tumorigenesis of NSCLC, we established xenograft tumor models using nu/nu mice. To minimize the number and suffering of the mice, five mice per group were used in this experiment, and the tumor volume was strictly monitored. Xenograft tumors established with NSCLC cells overexpressing TIPE3 grew faster than those established with control NSCLC cells, leading to increases in tumor size and weight. Immunohistochemistry showed that TIPE3, especially plasma membrane-localizing TIPE3, was obviously increased in the tumor tissue sections from mice received NSCLC cells overexpressing TIPE3 compared with those from mice received control NSCLC cells. These data provide direct evidences for the pro-tumorigenesis of human TIPE3 in NSCLC, and also emphasize the importance of subcellular location for TIPE3 function. More detailed mechanisms remain to be further investigated.

Notably, the essential role of plasma membrane-localizing TIPE3 in promoting cell growth and migration was further clarified in a transient overexpression system using plasmid transfection. In this system, either long or short TIPE3 transfected by plasmids inhibited the growth and migration of NSCLC cells, producing opposite effects to lentivirus transfection system; interestingly, after transient expression, TIPE3 was mainly localized in cytoplasm. These observations confirmed that different subcellular locations caused differential effects of human TIPE3 on the tumorigenesis of NSCLC. Plasma membrane-localizing TIPE3 drove a promotive effect on cell proliferation and migration of NSCLC cells, while cytoplasmic TIPE3 led to an inhibitory effect. Fayngerts et al. reported that N-terminal region was essential for the effects of TIPE3 on cell growth and survival, depletion of N-terminal region elicited a negative effect [8]. In the present study, TIPE3 with N-terminal flag in transient plasmid transfection system was detained in cytoplasm, while TIPE3 with C-terminal flag in stable lentivirus transfection system was orientated to plasma membrane. Since N-terminal domain, which is involved in signal cleavage, protein folding and modifications like glycosylation, is critical during protein targeting, translocation and insertion into membrane [24, 25], a very likely explanation for the discrepant effects of exogenous human TIPE3 is that flag at N-terminal domain influences the membrane location and then the function of TIPE3. Murine TIPE3 has been demonstrated to shuttle two lipid second messengers phosphatidylinositol 4,5-bisphosphate [PtdIns(4,5)P2], phosphatidylinositol 3,4,5-trisphosphate [PtdIns(3,4,5)P3] and increase their levels in plasma membrane [8]. Membrane PtdIns(3,4,5)P3 can recruit its effector protein molecules like AKT from cytoplasm to membrane surface to activate, stabilize, and propagate downstream signaling cascades, thus mediating a variety of physiological processes including cell survival and growth [26–29]. So, it would be necessary for human TIPE3 to orientate in plasma membrane to exert its promotive effect on NSCLC tumorigenesis. As for cytoplasmic TIPE3 binding with lipids, failure to target membrane may lead to decreases in membrane PtdIns(3,4,5)P3 and then the activation of downstream signals, thus mediating the inhibitory effect on NSCLC tumorigenesis. Therefore, appropriate strategies and detailed mechanisms for the application of TIPE3 in NSCLC therapy remain to be further investigated.

## Conclusion

Collectively, we provide evidences that human TIPE3 promotes the growth and migration of NSCLC cells depending on its localization in plasma membrane, whereas cytoplasmic TIPE3 exerts a negative function, thereby proposing TIPE3 as a potential target for NSCLC therapy.

## Additional file

Additional file 1: Comparison of long and short isoforms of human. TIPE3 (PDF 192 kb)

## Abbreviations

FBS: Fetal bovine serum
NSCLC: Non-small cell lung cancer
PtdIns (3,4,5)P3: Phosphatidylinositol 3,4,5-trisphosphate
PtdIns (4,5)P2: Phosphatidylinositol 4,5-bisphosphate
TH: TIPE2 homology
TIPE (TNFAIP8): Tumor necrosis factor-α-induced protein 8
