# Supplementary material for: TIPE3 differentially modulates proliferation and migration of human non-small-cell lung cancer cells via distinct subcellular location
Source: BMC Cancer. 2018 Mar 6;18:260. doi: 10.1186/s12885-018-4177-0 (PMC5840720; doi:10.1186/s12885-018-4177-0)
Supplement: Supplementary file 1 — Comparison of long and short isoforms of human. TIPE3 (PDF 192 kb) [file 12885_2018_4177_MOESM1_ESM.pdf]

Supplementary Material

Comparison of long and short isoforms of human TIPE3

```
long_TIPE3.seq     MGKPRQNPSTLVSTLCEAEPKGKLWVNGYAGTQGTRDATL     40
short_TIPE3.seq    ........................................      0

long_TIPE3.seq     QTRLIPLSFHLQRGKGLAAPLSALSAPRLPERPADGRVAV     80
short_TIPE3.seq    ........................................      0

long_TIPE3.seq     DAQPAARSMDSDSGEQSEGEPVTAAGPDVFSSKSLALQAQ    120
short_TIPE3.seq    ........MDSDSGEQSEGEPVTAAGPDVFSSKSLALQAQ     32

long_TIPE3.seq     KKILSKIASKTVANMLIDDTSSEIFDELYKVTKEHTHNKK    160
short_TIPE3.seq    KKILSKIASKTVANMLIDDTSSEIFDELYKVTKEHTHNKK     72

long_TIPE3.seq     EAHKIMKDLIKVAIKIGILYRNNQFSQEELVIVEKFRKKL    200
short_TIPE3.seq    EAHKIMKDLIKVAIKIGILYRNNQFSQEELVIVEKFRKKL    112

long_TIPE3.seq     NQTAMTIVSFYEVEYTFDRNVLSNLLHECKDLVHELVQRH    240
short_TIPE3.seq    NQTAMTIVSFYEVEYTFDRNVLSNLLHECKDLVHELVQRH    152

long_TIPE3.seq     LTPRTHGRINHVFNHFADVEFLSTLYSLDGDCRPNLKRIC    280
short_TIPE3.seq    LTPRTHGRINHVFNHFADVEFLSTLYSLDGDCRPNLKRIC    192

long_TIPE3.seq     EGINKLLDEKV                                 291
short_TIPE3.seq    EGINKLLDEKV                                 203
```
